# Does adjunctive hemoadsorption provide benefit in the management of ischemia–reperfusion syndrome following near-drowning? A case report

**DOI:** 10.3389/fmed.2024.1341156

**Published:** 2024-04-03

**Authors:** Pedja Kovacevic, Sasa Dragic, Milka Jandric, Danica Momcicevic, Vedrana Malesevic, Tijana Kovacevic, Marijana Matejic-Spasic, Tanja Knezevic, Biljana Zlojutro

**Affiliations:** ^1^Medical Intensive Care Unit, University Clinical Centre of the Republic of Srpska, Banja Luka, Bosnia and Herzegovina; ^2^Faculty of Medicine, University of Banja Luka, Banja Luka, Bosnia and Herzegovina; ^3^CytoSorbents Europe GmbH, Berlin, Germany

**Keywords:** drowning, ischemia–reperfusion injury, ECMO, hemoadsorption, CytoSorb, case report, near-drowning

## Abstract

Drowning remains a significant global health concern, claiming over 300,000 lives annually, with a disproportionate impact on young individuals in low-and middle-income countries. Conventional mechanical ventilation, while common, falls short in addressing the hypoxemia and hypercapnia often observed in severe near-drowning cases. Veno-venous extracorporeal membrane oxygenation (vvECMO) emerges as a critical intervention for cardiopulmonary failure post-drowning. This case report delves into the pivotal role of ischemia–reperfusion injury (IRI) in a near-drowning-related pathology. Following the initial insult, reoxygenation exacerbates the inflammatory cascade, resulting in a surge of pro-inflammatory mediators. In this context, CytoSorb^®^, a hemoadsorption cartridge, demonstrates promise by effectively removing these mediators from circulation. This report outlines its application in a critically ill adolescent patient who experienced near-drowning, presenting a compelling case for CytoSorb as an adjunctive therapy in managing IRI-induced hyperinflammation.

## 1 Introduction

According to the World Health Organization (WHO), more than 300,000 deaths occur each year as a result of unintentional drowning, making it a significant cause of preventable mortality and morbidity worldwide. While contemporary literature lacks a unified consensus on the definition of drowning, to present this case, the term “near-drowning” is deemed most suitable. This designation suggests that, after experiencing suffocation, the victim has survived for a minimum of 24 h following submersion in water (1, 2). Approximately 90% of these fatalities occur in low-and middle-income countries, with half of the victims being less than 25 years old (3). Drowning occurs when water is aspirated into the lungs, causing damage to surfactant, disrupting the alveolar-capillary membrane, and resulting in the formation of alveolar edema, which resembles a localized acute respiratory distress syndrome (ARDS)-like syndrome (4). These events can lead to circulatory dysfunction and cardiac arrest. A significant number of near-drowning patients experience hypoxia, characterized by a PaO_2_/FiO_2_ ratio < 300 mmHg (5). Conventional mechanical ventilation is typically employed in patients with severe ARDS following near-drowning. However, this approach may not adequately address hypoxemia or hypercapnia in these individuals, and it could potentially worsen pulmonary damage due to barotrauma and high levels of inspired oxygen (6). The implementation of veno-venous extracorporeal membrane oxygenation (vvECMO) is crucial in tackling the alarmingly low survival rates observed in patients with cardiopulmonary failure following drowning (7). Ultimately, treating lung injury and reversing hypoxia are essential components of effectively managing cases of near-drowning (8, 9). Ischemia–reperfusion injury (IRI), as a result of reoxygenation following hypoxia, commonly initiates the generation of reactive oxygen species and prompts the release of various signaling molecules, including pro-and anti-inflammatory mediators: interleukin (IL)-6, IL-8, IL-10, and tumor necrosis factor (TNF)-α (10, 11). If this acute phase advances to a generalized “cytokine storm” and becomes dysregulated due to the imbalance between pro-and anti-inflammatory mechanisms, the likelihood of a positive outcome dramatically decreases. IL-6, the most extensively studied cytokine, is frequently used as a benchmark for (hyper) inflammation due to its accessible detection test and established association with outcomes in critically ill patients (12). Recently, hemoadsorption of cytokines has been introduced as an adjunctive treatment option in the management of dysregulated immune response in critically ill (13). The CytoSorb hemoadsorption column consists of coated polymer beads that capture hydrophobic molecules with a molecular weight of up to approximately 60 kDa (cytokines, bilirubin, bile acids, free hemoglobin, myoglobin, bacterial exotoxins, etc.) by surface adsorption and size exclusion (12, 14). Data on the effect of CytoSorb in IRI *per se* are scarce. Therefore, this case report aims to describe the possible positive effects of CytoSorb in the setting of ischemia–reperfusion injury following the near-drowning of a critically ill adolescent patient.

## 2 Case presentation

A previously healthy 16-year-old male patient, 183 cm tall and weighing approximately 83 kg body-mass index (BMI) of 24.8, was admitted to the Emergency Room of the University Clinical Center. He presented with altered consciousness and respiratory insufficiency following a drowning incident in fresh water. Upon admission, the patient was unconscious, with a Glasgow Coma Scale (GCS) score of 4, symmetrical and reactive pupils of medium width, low peripheral oxygen saturation, tachycardia, but normal blood pressure. Blood gas analysis (ABG) at admission showed a pH of 6.69, pCO_2_ 8.61 kPa, pO_2_ 6.37 kPa, HCO_3_ 8.5 mmol/L, base excess (BE) -13.8 mmol/L, glucose 4.7 mmoL/L, and lactate 19.3 mmol/L (Table 1). Given the altered consciousness and inability of proper airway protection, the patient was immediately intubated and placed on controlled mechanical ventilation. As part of the diagnostic evaluation procedure, a computed tomography (CT) scan was performed according to the polytrauma protocol (CT scan of the head, neck, chest, abdomen, and pelvis), revealing clear fluid in the right maxillary and sphenoid sinuses. Multiple, confluent areas of consolidation in the lung parenchyma of both lungs were found, primarily indicative of pulmonary edema due to aspiration. The esophagus was dilated and filled with fluid throughout the thoracic segment. Additionally, hepatomegaly and steatosis were observed. The abdomen was noticeably distended, extending above the sternum level, and exhibited a tympanic response to percussion. Additionally, no sounds were detected upon auscultation of the abdomen. The CT revealed signs consistent with paralytic ileus, primarily characterized by gas accumulation and partially by fluid. Upon consultation with the on-duty intensivist, admission to a Medical Intensive Care Unit (MICU) was indicated. In MICU, the patient was still hemodynamically unstable and hypotensive with a blood pressure of 85/60 mmHg, tachycardic with a heart rate of 120/min, and a subfebrile temperature of 37.5°C. Diffuse moist inspiratory-expiratory crackles were detected in the lung parenchyma while cardiac function proved impaired. The abdomen was tense, with no organomegaly and audible peristalsis. No edema was observed in the extremities. A urinary catheter was placed and 700 mL of hematuric urine was collected.

**Table 1:** Laboratory and clinical parameters throughout the clinical course.

Parameter	Reference range	Unit	Day 1*	Day 2	Day 3*	Day 4	Day 5	Day 6	Day 7	Day 8	Day 9	Day 10	Day 11	Day 12	Day 13	Day 14	Day 15
Leucocytes	4.40–11.6	[10^3^/μl]	14.73	12.95	14.95	16.06	11.23	8.66	8.02	7.61	7.78	14.14	/	11.4	8.07	8.64	6.18
Platelets	178–420	[10^3^/μl]	285	107	72	67	76	90	100	134	168	190	/	195	224	233	239
Creatinine	14–68	[μmol/l]	110	88	79		55	51	44	/	/	/	/	/	/	/	54
AST	0–50	[U/l]	203	191	400	153	63	/	57	/	27	/	/	/	/	/	30
ALT	0–50	[U/l]	97	85	455	339	199	/	124	/	81	/	/	/	/	/	/
Bilirubin total	5–21	[mg/dl]	5.4	35.7	14.2	17.4	15.4	/	15.7	/	14.3	/	/	/	/	/	/
Bilirubin direct	0–3	[mg/dl]	/	25.9	8.5	9.6	7.2	/	5.6	/	5.1	/	/	/	/	/	/
CRP	0–5	[mg/l]	0.7	16.9	125.4	83.0	43.4	26.9	20.1	/	9.6	/	/	5.2	/	/	/
Procalcitonin	<0.5	[ng/ml]	0.020	/	17.48	7.25			0.810	/	/	/	/	0.24	/	/	/
Norepinephrine		[μg/kg/min]	0.8	0.25	0.2	0.05	/	/	/	/	/	/	/	/	/	/	/
Lactate	0–2	[mmol/l]	19	3.33	1.64	1.47	1.46	0.92	0.76	0.78	0.76	0.95	/	/	/	/	/
Temperature		[°C]	37.5	36.1	36.4	37.6	38	37.7	37.4	37.1	36.6	36.6	36.7	36.6	36.7	36.7	36.6
Interleukin-6	0–4.4	[pg/ml]	10249	764	/	14.1	7.35	8.37	8.90	/	/	/	/	/	/	/	/
pH	7.35–7.45		6.9	7.26	7.39	7.37	7.49	7.47	7.40	7.38	7.39	7.41	/	/	/	/	/
FiO_2_	0.21		1.0	0.8	0.35	0.5	0.4	0.3	0.3	0.25	0.25	0.21	0.21	0.21	0.21	0.21	0.21
PEEP	5	[mmHg]	26	20	14	8	10	10	8	/	/	/	/	/	/	/	/
P/F ratio	>300	[mmHg]	40	39	30	97	80	81	107	146	97	179	/	/	/	/	/
Ventilation mode			PRVC	PRVC	PRVC	PRVC	PRVC	PRVC	HFNC	HFNC	HFNC	HFNC	/	/	/	/	/

AST – aspartate aminotransferase; ALT – alanine aminotransferase; CRP – C-reactive protein; PEEP – Positive end-expiratory pressure; PRVC – Pressure-regulated volume; HFNC; high flow nasal cannula control. *Indicator of CytoSorb treatment start (1st – 3 h + 2nd – 12 h on Day 1, 3rd – 12 h on Day 3).

**Figure 1:**
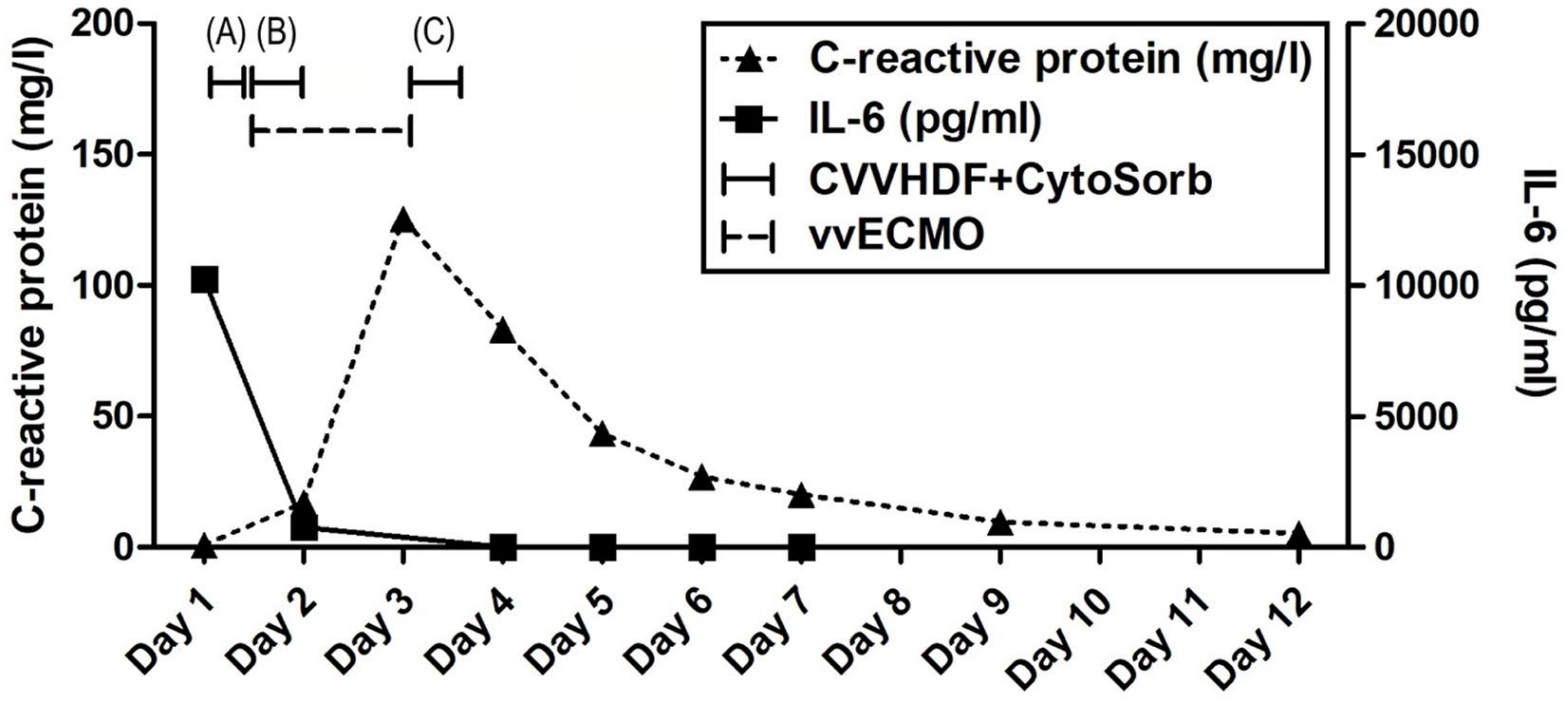
Evolution of inflammation throughout the clinical course. Timeline of hemoadsorption intervention: (A) 1st CytoSorb – 3 h-treatment on the first day of hospitalization, just before vvECMO commenced, therapeutic; (B) 2nd CytoSorb – 12 h-treatment parallel to vvECMO start, therapeutic; (C) 3rd CytoSorb – 12 h treatment immediately after weaning from vvECMO (decannulation), prophylactic.

**Figure 2:**
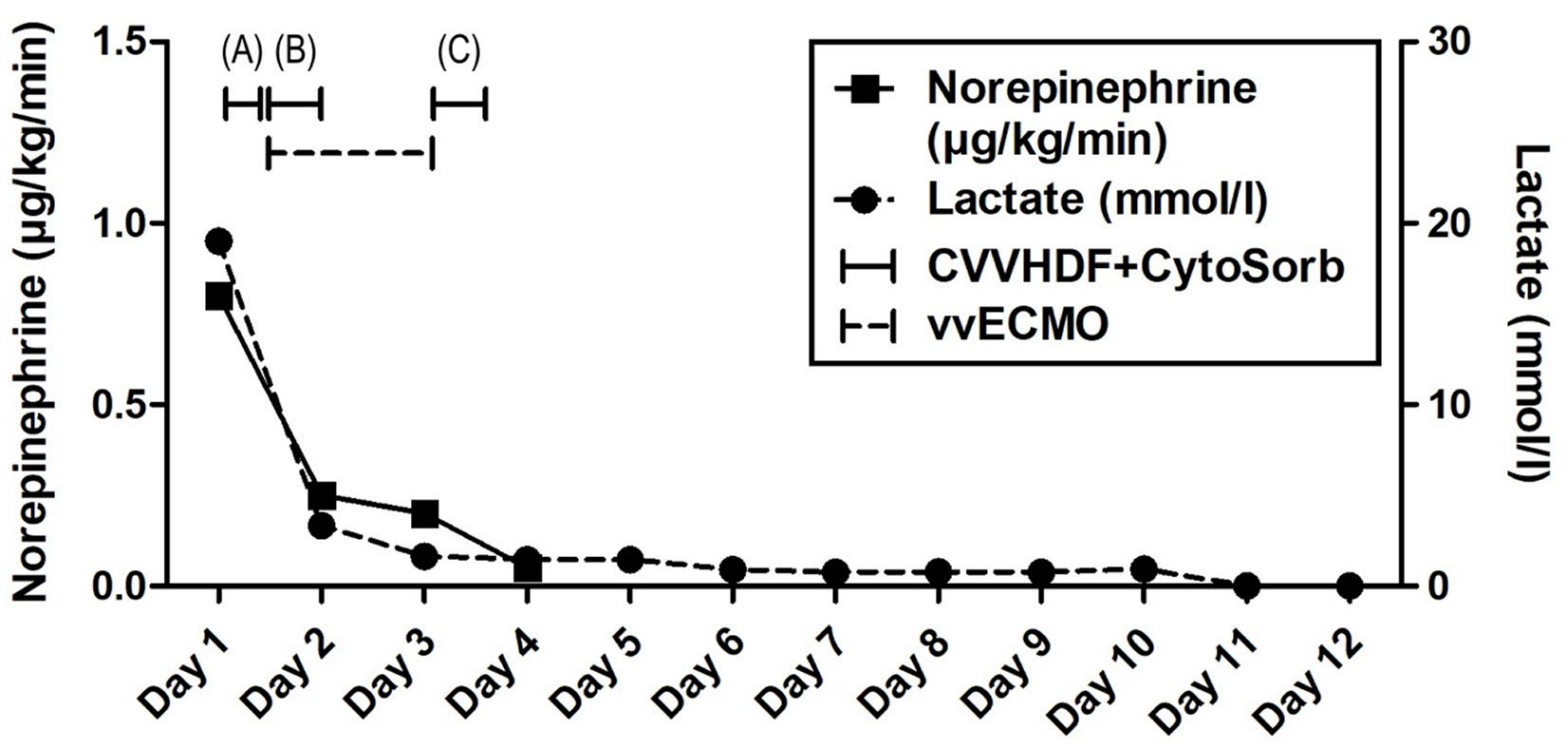
Evolution of hemodynamic parameters throughout the clinical course. Timeline of hemoadsorption intervention: (A) 1st CytoSorb – 3 h-treatment on the first day of hospitalization, just before vvECMO commenced, therapeutic; (B) 2nd CytoSorb – 12 h-treatment parallel to vvECMO start, therapeutic; (C) 3rd CytoSorb – 12 h treatment immediately after weaning from vvECMO (decannulation), prophylactic.

Advanced respiratory as well as hemodynamic monitoring was commenced. Under maximum respiratory support settings with the fraction of inspired oxygen (FiO_2_) set to 1.0, a peripheral oxygen saturation (SpO_2_) of 95% could be achieved. Immediate interventions further included continued controlled mechanical ventilation, continuous infusions of sedation and muscle relaxants, as well as continuous vasopressor support (norepinephrine 0.8 μg/kg/min) due to hypotension. Immediately after admission, a therapeutic protocol was instituted, comprising an infusion of crystalloid and colloid solutions, prophylaxis against stress ulcers, corticosteroids, and anti-edema therapy. Additionally, continuous heparin infusion was adjusted every 6 h based on aPTT (activated Partial Thromboplastin Clotting Time) values. Intravenous administration of empirical triple antibiotic therapy in the following dosage regimen was initiated: ceftriaxone 2 g bid (*bis in die* - twice a day), clindamycin 600 mg tds (*ter in die* – three times a day), and vancomycin 1 g bid. Due to the onset of a hyperinflammatory condition marked by extremely elevated levels of IL-6 (>10,000 pg/mL), metabolic acidosis, and acute kidney injury (AKI), a central venous catheter (CVC) was inserted. Continuous veno-venous hemodiafiltration (CVVHDF) using Fresenius multiFiltrate Ultraflux AV600S was initiated. This treatment was augmented by adjunctive hemoadsorption therapy (CytoSorb®, CytoSorbents Inc., Princeton, NJ, USA), integrated into pre-hemofilter mode while administering unfractionated heparin for anticoagulation. Inflammation parameters were initially assessed from the patient’s serum before and after the therapeutic application of the adsorber on the first day, followed by daily monitoring thereafter (Figure 1). After 3 h of combined CVVHDF+CytoSorb treatment, the patient’s condition deteriorated further, with worsening respiratory function and the onset of pulmonary edema, evident by the foamy liquid in the endotracheal tube. Peripheral saturation dropped from 94 to 79%, despite the initially set positive end-expiratory pressure (PEEP) values of 16. Upon auscultation of the lungs, audible phenomena suggestive of pulmonary edema were observed. A recruitment maneuver was promptly performed, stabilizing the pulmonary edema, and the target PEEP value was adjusted to 26. Due to the patient’s preparation for vvECMO, the CVVHDF+CytoSorb treatment was discontinued. Following family consent, the patient was connected to vvECMO therapy due to the development of severe ARDS. Blood purification therapy with CVVHDF+CytoSorb was re-instituted, with a break of 2 h, and it ran for another 12 h with the new adsorber in place. The patient was ventilated in a protective “lung rest” mode complemented by vvECMO. In order to improve ventilation and fluid redistribution, the patient was placed in prone position. Isolates from tracheal aspirates confirmed *Acinetobacter calcoaceticus-baumannii* complex and adenovirus which was treated with colistin in the first dose of 9 MIU i.v., followed by 4.5 MIU 2×1 i.v. for 10 days consecutively. Following the second CVVHDF+CytoSorb 12-h interval treatment, the patient’s condition gradually improved. A sharp decrease in IL-6 levels (from 10,249 to 764 pg/mL) with clear time association with combined 15 h of hemoadsorption therapy indicated attenuation of hyperinflammation, together with hemodynamic stabilization marked by drop in norepinephrine requirements (from 0.8, at the time of admission, to 0.25 μg/kg/min, on the second ICU day) and lactate levels (from 19.3 to 3.3 mmol/L) through the second day of hospitalization. The need for a vasopressor (norepinephrine) terminated on day 4 of the ICU treatment (Figures 1, 2; Table 1).

The vvECMO treatment was terminated on day 3 following the stabilization of hemodynamic parameters, marked by further decrease in vasopressor dosage. As per our internal protocol for post-ECMO weaning prevention of hyperinflammation, immediately after decannulation, the patient was connected to the extracorporeal circuit for a prophylactic 12-h hemoadsorption treatment with one CytoSorb adsorber. Respiratory function showed improvement with a gradual reduction of PEEP, alongside restoration of diuresis and renal function. Acid–base balance was reestablished through the normalization of lactate levels, and inflammatory biomarkers IL-6 and C-reactive protein (CRP) (Figures 1, 2; Table 1). Subsequently, the patient was successfully extubated on the fifth day of hospitalization. During the following days, the levels of inflammatory parameters remained within normal range, indicating the absence of systemic inflammation (Figure 1; Table 1). Sinus arrhythmia was seen on electrocardiogram (ECG) while echocardiography showed preserved cardiac function. The patient’s fluid and electrolyte balance were managed, and nutritional support was provided. Hemodialysis and blood product transfusion were also administered as needed. During the next 7 days, the patient’s condition continued to improve, with stable vital signs, adequate consciousness, independent breathing, and sufficient diuresis while making good overall clinical progress. Therapy was adjusted based on laboratory and microbiological results, and daily monitoring continued.

On day 12, the patient’s clinical course was complicated by the development of CVC-related venous thrombosis, which was managed conservatively. Other than that, further clinical course was uneventful. All the interventions, including adjunctive hemoadsorption, were well tolerated. No device-related adverse events were observed. The patient was discharged from the hospital after 15 days, at which point all monitored laboratory, lung function, and other values were returned within reference ranges (Table 1). Recommendations for outpatient care, including anticoagulation therapy (enoxaparin 0.8 subcutaneously bid) and follow-up, were provided.

## 3 Discussion

The presented case underscores the critical role of ischemia–reperfusion injury (IRI) in the context of near-drowning-related pathology and its management using a combination of extracorporeal techniques, including vvECMO, CVVHDF, and CytoSorb hemoadsorption. The combination of CytoSorb and CVVHDF within the context of vvECMO was well tolerated, with no reported device-related technical complications or adverse events during or after the treatment. The patient’s presentation was consistent with the typical consequences of near-drowning, including the aspiration of water leading to pulmonary edema and impaired gas exchange. The patient required immediate intubation and mechanical ventilation, which played a pivotal role in ensuring oxygenation. The lack of oxygen intake coupled with compromised peripheral circulation, however, resulted in tissue ischemia and severe hypoxia including the buildup of lactate and other toxins, and a clinical presentation of severe ARDS secondary to near-drowning. Following resuscitation, the reperfusion process came with its own set of challenges, as toxins that were previously sequestered in tissues were released into the systemic circulation, triggering a massive inflammatory response. This reperfusion-induced inflammation phenomenon is well-documented in the context of IRI after various insults, including near-drowning (4, 15). The massive inflammatory response observed in this patient, as evidenced by whoppingly increased IL-6 levels, is in line with the activation of a variety of signaling molecules and the generation of reactive oxygen species during IRI (15). Various extracorporeal technologies were employed throughout the course of the patient’s treatment to address the detrimental effects of ARDS, IRI, and the inflammatory response. Following the implementation of standard therapeutic measures, adjunctive hemoadsorption therapy with CytoSorb alongside CVVHDF was introduced during the early phase of the patient’s deterioration, targeting the post-resuscitation inflammatory surge.

The use of precision medicine in critical care allows for customized interventions that target specific pathophysiological mechanisms in the appropriate patient populations. It has been shown that hypercytokinemia, particularly of IL-6, is associated with organ dysfunction, inadequate treatment response, and a worsened prognosis in sepsis. Sepsis occurs when the initially appropriate host response to infection becomes amplified and subsequently dysregulated, leading to an imbalance between pro-inflammatory and anti-inflammatory responses. CytoSorb facilitates a quicker restoration of balance and immune homeostasis by attenuating both pro-and anti-inflammatory responses. As such, it may have a role as rescue therapy in a particular subgroup of patients with refractory septic/vasoplegic shock, hyperlactatemia, multiorgan failure (MOF), and very high hypercytokinemia (16). For optimal results, it is crucial to initiate hemoadsorptive treatment within 12 h of diagnosing vasoplegic shock, and continue until hemodynamic stability is achieved, replacing adsorbers as needed (17). The decision to employ CytoSorb in the treatment of our patient was completely aligned with the aforementioned criteria – massive hypercytokinemia (IL-6 > 10,000 pg/mL) and lactatemia (>19 mmol/L), vasoplegic shock (norepinephrine dose of 0.8 μg/kg/min), and MOF (severe ARDS, AKI requiring CRRT, paralytic ileus). While initially not yielding significant clinical improvements, a subsequent administration of the second CytoSorb adsorber, following the initiation of vvECMO, was associated with a notable improvement in the patient’s condition, including a substantial decrease in IL-6 levels, a reduced requirement in vasopressor support as well as decreasing parameters of hypoxia and metabolic acidosis (Figures 1, 2; Table 1). The development of severe ARDS triggered the need for more aggressive respiratory and hemodynamic support. As such, vvECMO provided temporary cardiac and respiratory support, allowing time for lung recovery, and minimizing further damage due to mechanical ventilation. With respect to combined CVVHDF and hemoadsorption therapy, this positive reaction suggests that CytoSorb may have effectively attenuated the hyperinflammatory response. Of particular interest is the third administration of CytoSorb, which was intended for prophylactic use aiming to prevent post-decannulation systemic hyperinflammation, a pivotal consideration during the transition from vvECMO to conventional respiratory support. Numerous complications may occur after weaning from vvECMO, compromising the success of the treatment, sometimes even to the extent of being life-threatening. Complex and multi-faceted innate inflammatory responses to the artificial materials of the extracorporeal circuit contribute to the development of systemic hyperinflammation (18). This strategic intervention underscores the potential utility of CytoSorb not only as a reactive treatment but also as a preventive measure against complications arising from inflammation. Recent research within a highly standardized and reproducible human *in vivo* model of systemic inflammation and immunological tolerance induced by administration of bacterial lipopolysaccharide (LPS), it was proven that CytoSorb hemoperfusion effectively attenuated circulating cytokine concentrations during systemic inflammation in humans, whereas it did not affect long-term immune function (19). In this study, but also in a few others (11, 20), CytoSorb-adsorbed molecules belonged to both pro-inflammatory (TNF-α, IL-2, IL-6, IL-8, IL-18, MCP-1, MIP-1α)^1^ and anti-inflammatory mediators (IL-4, IL-10). These findings further support the notion of the benefit of rebalancing the inflammatory response with non-selective cytokine removal rather than focusing solely on pro-inflammatory mediators. In our case, CytoSorb might have also reduced levels of other inflammation triggers and mediators, as well as products of injury and organ damage, however these were not measured. Therefore, CytoSorb therapy may be of benefit in conditions characterized by dysregulated host response to infection and IRI-induced hyperinflammation. By the study conducted in 23 patients undergoing extracorporeal life support (ECLS) therapy and CytoSorb hemoadsorption, the initiation of CytoSorb treatment was prompted by the necessity for renal replacement therapy (CRRT) and the presence of one or more critical factors, including severe hyperinflammatory activation, profound reperfusion injury, extended cardiopulmonary bypass durations with subsequent post-cardiotomy low cardiac output, and an unresponsive vasoplegic response with rapidly progressing organ dysfunction (21). Significant reductions were observed in key inflammatory markers such as IL-6. Hemodynamic stability was achieved, marked by a notable decrease in vasopressor requirements. Importantly, ECLS flow rates were effectively maintained, and parameters such as lactate levels, pH, and BE were successfully stabilized and normalized during and after the treatment phase. These fundings are in line with the observations described in this case report. Moreover, similar outcomes were reported in a propensity-score matched study of veno-arterial (va)ECMO patient population where adjunctive usage of CytoSorb was associated with accelerated recovery of multiorgan and microcirculatory dysfunction, mitigated inflammatory response, less bleeding complications, and lower risk for early mortality in comparison with controls (22).

The present case report has limitations related to a single case observation and no control group. In addition, the patient underwent multiple concomitant pharmacological and blood purification treatments, and therefore to none specifically the survival and the clinical course without *sequelae* can be attributed. A certain anti-inflammatory effect of heparin has been described in the literature (23), and the causative antimicrobial therapy is undoubtedly essential in sepsis treatment, as well as vasopressor support for vasoplegic shock. Nonetheless, controlled studies (24–26) have shown that adjunctive hemoadsorption significantly reduced levels of inflammatory mediators and improved outcomes in patients with comparable critical conditions and similar concomitant therapeutic protocols.

The boy’s father and legal representative in this case, is a healthcare worker employed as a perfusionist in our MICU. He is one of the first perfusionists in our country, which has created a significant burden in the treatment process itself. On the other hand, the father agreed to all off-label therapeutic modalities, including a prophylactic hemoadsorption post-vvECMO weaning, and showed boundless gratitude for the selfless effort of the whole team, the innovation of the therapy protocols, and the positive outcome of the treatment.

In conclusion, the presented case of a critically ill adolescent patient who experienced near-drowning highlights the intricate interplay between ischemia, reperfusion, and the subsequent inflammatory response. The combined use of various extracorporeal technologies, including vvECMO, CRRT, and CytoSorb, played a crucial role in managing the patient’s condition. The strategic administration of CytoSorb at different time points underscores its potential as a therapeutic intervention in attenuating the hyperinflammatory response. This case therefore adds to the existing literature on CytoSorb’s potential efficacy in mitigating dramatic clinical presentations associated with excessive cytokine release and hypoxia. Moreover, it provides valuable insights into the management of complex cases involving IRI. However, more research is needed to fully understand its optimal application in the clinical context of near-drowning-related ischemia–reperfusion injury.

## Data availability statement

The raw data supporting the conclusions of this article will be made available by the authors, without undue reservation.

## Ethics statement

Ethical approval was not required for this study involving human samples in accordance with the local legislation and institutional requirements because of the retrospective nature of this case report. Written informed consent for participation in this study was provided by the participant’s legal guardian/next of kin. Written informed consent was obtained from the minor’s legal guardian/next of kin, for the publication of any potentially identifiable images or data included in this article.

## Author contributions

PK: Writing – original draft, Writing – review & editing. SD: Data curation, Writing – review & editing. MJ: Conceptualization, Data curation, Writing – review & editing. DM: Data curation, Writing – review & editing. VM: Investigation, Writing – review & editing. TKo: Formal Analysis, Validation, Writing – review & editing. MM-S: Writing – review & editing. TKn: Data curation, Writing – review & editing. BZ: Writing – review & editing.

## Footnotes

^1^MCP-1 - monocyte chemoattractant protein-1, MIP-1α- macrophage inflammatory protein-1 alpha.
